# Phase I dose-escalation study of procaspase-activating compound-1 in combination with temozolomide in patients with recurrent high-grade astrocytomas

**DOI:** 10.1093/noajnl/vdad087

**Published:** 2023-07-19

**Authors:** Matthias Holdhoff, M Kelly Nicholas, Richard A Peterson, Stefania Maraka, Li C Liu, James H Fischer, Jeffrey S Wefel, Timothy M Fan, Tracy Vannorsdall, Meredith Russell, Michaella Iacoboni, Theodore M Tarasow, Paul J Hergenrother, Arkadiusz Z Dudek, Oana C Danciu

**Affiliations:** Department of Oncology, Johns Hopkins University School of Medicine, The Sidney Kimmel Comprehensive Cancer Center at Johns Hopkins, Baltimore, Maryland, USA; Department of Neurology and Rehabilitation, University of Illinois at Chicago, Chicago, Illinois, USA; HealthPartners Institute, Regions Cancer Care Center, St. Paul, Minnesota, USA; Department of Neurology and Rehabilitation, University of Illinois at Chicago, Chicago, Illinois, USA; Division of Epidemiology and Biostatistics, School of Public Health, University of Illinois at Chicago, Chicago, Illinois, USA; Department of Pharmacy Practice, College of Pharmacy, University of Illinois at Chicago, Chicago, Illinois, USA; Department of Neuro-Oncology, MD Anderson Cancer Center, Houston, Texas, USA; Vanquish Oncology, Inc., Champaign, Illinois, USA; Department of Veterinary Clinical Medicine, University of Illinois, Urbana-Champaign, Illinois, USA; Cancer Center at Illinois, Urbana-Champaign, Illinois, USA; Department of Psychiatry and Behavioral Sciences, Johns Hopkins University School of Medicine, Baltimore, Maryland, USA; Clinical Trials Office, University of Illinois Cancer Center, University of Illinois at Chicago, Chicago, Illinois, USA; Department of Oncology, Johns Hopkins University School of Medicine, The Sidney Kimmel Comprehensive Cancer Center at Johns Hopkins, Baltimore, Maryland, USA; Vanquish Oncology, Inc., Champaign, Illinois, USA; Institute for Genomic Biology, University of Illinois, Urbana-Champaign, Illinois, USA; Vanquish Oncology, Inc., Champaign, Illinois, USA; Cancer Center at Illinois, Urbana-Champaign, Illinois, USA; Institute for Genomic Biology, University of Illinois, Urbana-Champaign, Illinois, USA; Department of Chemistry, University of Illinois, Urbana-Champaign, Illinois, USA; HealthPartners Institute, Regions Cancer Care Center, St. Paul, Minnesota, USA; Vanquish Oncology, Inc., Champaign, Illinois, USA; Division of Hematology, Oncology and Transplantation, University of Minnesota, Minneapolis, Minnesota, USA; Clinical Trials Office, University of Illinois Cancer Center, University of Illinois at Chicago, Chicago, Illinois, USA; Division of Hematology/Oncology, Department of Medicine, University of Illinois at Chicago, Chicago, Illinois, USA

**Keywords:** astrocytoma, glioblastoma, PAC-1, Procaspase-activating compound-1, recurrence, temozolomide

## Abstract

**Background:**

Procaspase-3 (PC-3) is overexpressed in various tumor types, including gliomas. Targeted PC-3 activation combined with chemotherapy is a novel strategy for treating patients with high-grade gliomas, with promising preclinical activity. This study aimed to define safety and tolerability of procaspase-activating compound-1 (PAC-1) in combination with temozolomide (TMZ) for patients with recurrent high-grade astrocytomas.

**Methods:**

A modified-Fibonacci dose-escalation 3 + 3 design was used. PAC-1 was administered at increasing dose levels (DL; DL1 = 375 mg) on days 1–21, in combination with TMZ 150 mg/m^2^/5 days, per 28-day cycle. Dose-limiting toxicity was assessed during the first 2 cycles. Neurocognitive function (NCF) testing was conducted throughout the study.

**Results:**

Eighteen patients were enrolled (13 GBM, IDH-wild type; 2 astrocytoma, IDH-mutant, grade 3; 3 astrocytoma, IDH-mutant, grade 4). Dose escalation was discontinued after DL3 (ie, PAC-1, 625 mg) due to lack of additional funding. Grade 3 toxicity was observed in 1 patient at DL1 (elevated liver transaminases) and 1 at DL 2 (headache). Two partial responses were observed at DL1 in patients with GBM, O^6^-methylguanine-DNA methyltransferase (MGMT) promoter methylated. Two patients had stable disease, and 11 experienced progression. NCF testing did not show a clear relationship between PAC-1 dose, treatment duration, and declines in NCF.

**Conclusions:**

Combination of PAC-1 and TMZ was well tolerated up to 625 mg orally daily and TMZ orally 150 mg/m^2^/5 days per 28-day cycle. The maximum tolerated dose was not reached. Further dose escalation of PAC-1 in combination with TMZ is advised before conducting a formal prospective efficacy study in this patient population.

**Key Points**

(1) PAC-1 and TMZ in recurrent high-grade astrocytoma were well tolerated at dose levels studied.
(2) Further study is needed to determine maximum tolerated dose and efficacy in prospective trials.

**Importance of the Study**

Novel treatments are urgently needed for the treatment of glioblastomas (GBM) and other high-grade gliomas. Procaspase-activating compound-1 (PAC-1) is a novel oral drug with promising preclinical data in these cancers. This study aimed to determine the MTD of PAC-1 in combination with temozolomide (TMZ) in recurrent high-grade astrocytomas to pave the way for efficacy studies with this combination in GBM and other primary brain cancers. Unfortunately, due to discontinuation of funding, the MTD could not be reached. Safety and tolerability could be determined up to dose level 3. Here we present the comprehensive clinical, pharmacokinetic, and neuropathologic data from our study that should serve as a platform for further study of PAC-1 and TMZ for these cancers.

Novel therapies are urgently needed to improve the outcome for patients with high-grade astrocytomas as limited effective treatments exist and as virtually all patients eventually die of their disease. Glioblastoma (GBM) is the most common primary brain cancer in adults. Initial treatment has not significantly changed since the introduction of temozolomide (TMZ) in 2005, and standard of care has remained treatment with radiation and TMZ in newly diagnosed patients, with or without tumor treatment fields in the adjuvant setting after completion of chemoradiation.^1^ Radiation and TMZ are also standard for newly diagnosed astrocytomas, IDH-mutant, World Health Organization (WHO) grade 3, formerly termed anaplastic astrocytoma.^2^ For patients that have been previously treated with standard radiation and TMZ but that had not progressed on TMZ, a TMZ re-challenge can be a reasonable treatment choice.

Caspase-3 and -7 are cysteine proteases that are involved in execution of apoptosis, programmed cell death that is critical to normal cell development in cells and higher organisms. Proteolytic conversion of procaspase-3 (PC-3) to caspase-3 is pivotal in the apoptotic cascade. PC-3 is found to be overexpressed in many cancers, suggesting a potential oncogenic role of PC-3, making this an attractive target for drug design.^3^ The compound PAC-1 was identified as a promising anticancer agent through screening of ~20 000 small molecules, for their ability to activate PC-3 in vitro and to induce apoptosis in cancer cell cultures.^4–6^ Multiple lines of research suggest the potential of PAC-1 for treatment of GBM: (1) Procaspase-3 is overexpressed in brain tumors, including GBM.,^7,8^ (2) PAC-1 is not a substrate of the P-glycoprotein efflux pump that prevents many drugs from reaching significant concentration in the brain.,^9^ (3)PAC-1 was found to significantly penetrate the blood-brain barrier in mice, consistent with cLogBB prediction models.,^10^ (4) PAC-1 shows promising activity against human glioma cell lines and in intracranial models in rodents, including syngeneic and xenograft models, as a single agent and in combination with TMZ., and^7,8^ (5) Finally, the PAC-1 development pathway involved extensive evaluation in pet dogs with cancer,^11,12^ including promising results in canine cancer patients (in combination with TMZ and radiation) with glioma, where all treated glioma dogs had responses, including one complete response.^8^ Also the PAC-1/TMZ combination was effective in treating pet dogs with meningioma, reducing tumor burden in all treated dogs.^13^ Hence, PAC-1 is an attractive compound for clinical testing in primary brain cancers, especially in combination with TMZ.

In a phase 1 dose-escalation study in patients with systemic solid tumors with single agent PAC-1, we determined a recommended phase 2 dose (RP2D) of oral PAC-1 at 750 mg/day, with response data that were found to warrant further clinical investigation, especially in neuroendocrine tumors.^14^ In a second component that is presented here, based on the hypothesis-generating preclinical data in gliomas, we investigated safety and tolerability of PAC-1 in combination with standard TMZ in the treatment of patients with high-grade astrocytoma that had recurred after initial standard therapy and that were candidates for repeat treatment with TMZ.

## Methods

### Patients

Patients 18 years or older with recurrent GBM or anaplastic astrocytoma, per 2016 WHO classification of cancers of the central nervous system, were eligible for participation in this multi-center trial (Clinical Trials.gov: NCT03332355). Patients were required to have (1) an Eastern Cooperative Oncology Group performance status of 0, 1, or 2, (2) measurable disease per Response Criteria in Neuro-Oncology (RANO), and (3) adequate hematologic, hepatic, and renal function. Patients had previously received standard radiation and TMZ as their initial therapy, but they had not previously progressed while on TMZ. The study was conducted according to International Conference on Harmonization of Good Clinical Practice guidelines and the principles of the Declaration of Helsinki. The protocol was approved by the Institutional Review Board/Ethics Committee at each study location. All patients provided written informed consent before enrollment.

### Study Treatment

Patients received oral PAC-1 at the assigned oral dose on days 1–21 of each 28-day cycle, and TMZ at 150 mg/m^2^ for 5 consecutive days starting on day 8 of each 28-day cycle.

### Study Design

Four dose levels (DLs) were planned for PAC-1 dose escalation: DL1, 375 mg daily; DL2, 500 mg daily; DL3, 625 mg daily; and DL4, 750 mg daily. TMZ was to be administered at the same dose and schedule at each of the 4 PAC-1 DLs.

Safety and tolerability of PAC-1 in combination with TMZ were determined using a modified-Fibonacci dose-escalation 3 + 3 design. Three patients were initially enrolled into each DL cohort, but additional patients were enrolled, as needed, to ensure that 3 patients completed 2 full treatment cycles and were evaluable for dose-limiting toxicity (DLT) assessment. Escalation to the next dose continued unless a patient experienced a DLT, at which time a cohort was expanded to up to 6 patients evaluable for DLT assessment and able to complete 2 full cycles of treatment. The definition of a DLT is detailed further in Supplementary Material.

### Evaluation of Toxicity and Response

Toxicity and adverse side effects were classified according to NCI’s Common Terminology Criteria for Adverse Events Version 4.0 (CTCAE v 4) and assessed on day 1 of each cycle. Magnetic resonance imaging was performed every 8 weeks of treatment to assess disease status. Standard clinical measures were used to assess response using RECIST version 1.1.

### Pharmacokinetics

Pharmacokinetics (PK) were assessed in each dose cohort for PAC-1 following doses administered on days 7 (without TMZ) and 12 (with TMZ) of the first cycle and TMZ on day 12 of cycle 1. Both drugs were administered under fasting conditions in the morning for PAC-1 and morning or evening for TMZ. To facilitate the PK study and amplify any PK interaction between drugs, the administration of TMZ was standardized to occur in the morning on cycle 1, day 12 two hours before PAC-1. Plasma samples were collected on the mornings of days 7 and 12 of cycle 1 prior to oral ingestion of TMZ (day 12 only), 1 hour (day 12) or immediately (day 7) before administration of PAC-1 and at 0.5, 1, 2, 4, 6, 8, 10, and 24 hours after ingestion of PAC-1 (both days). The PAC-1 and TMZ concentrations in plasma were determined by validated high-performance liquid chromatography and tandem mass spectrometry (LC-MS/MS) assays. Non-compartmental analysis of the PAC-1 and TMZ plasma concentration-time data was performed using WinNonlin version 8.1 (Certara. L.P., Princeton, NJ). The analysis occurred at steady state for PAC-1 and non-steady state conditions for TMZ. Parameters included: maximum plasma concentration (C_max_), time at C_max_ (T_max_), minimum plasma concentration at steady-state (C_ss,min_), area under the PAC-1 plasma concentration-time curve over the steady-state dosing interval (AUC_0-τ_,_ss_), area under the TMZ plasma concentration-time curve from time zero to infinity (AUC_0-∞_), oral clearance (CL/F), volume of distribution (V/F), and terminal phase half-life (t_1/2_).

### Neurologic Exams and NCF Testing

Neurocognitive function (NCF) testing was administered by a trained, certified member of the site study team (see online Supplementary Material for details of training). Per protocol, NCF testing was completed at baseline, day 1 of each cycle (except cycle 1 day 1), and 30 days after the final dose of PAC-1. A validated battery of cognitive tests that have been previously utilized in brain tumor clinical trials were administered in this trial to assess learning and memory (Hopkins Verbal Learning Test-Revised [HVLT-R]), verbal fluency (Controlled Oral Word Association), processing speed (Trail Making Test Part A [TMT-A]), and executive function (Trail Making Test Part B [TMT-B]).^15–17^

### Immunohistochemistry

Immunohistochemical (IHC) staining was performed on formalin-fixed, paraffin-embedded (FFPE) tumor tissues and is described in Supplementary Material.

### Statistical Analysis

The primary endpoint of this study was to establish tolerability of PAC-1 in combination with TMZ using a modified-Fibonacci dose-escalation 3 + 3 design. The maximum tolerated dose (MTD) would be defined as that dose of PAC-1 with DLT of less than 33% in first cycle of therapy or the first 2 cycles of therapy (neurological toxicity). Statistical considerations for the PK and NCF analysis are summarized in Supplementary Material. For clinical data, descriptive statistics were used to describe the study sample. Three clinical endpoints of interest, response (Stable Disease or Partial Response), progression-free survival, and overall survival times, were estimated using 95% confidence interval estimation. Bivariate associations between demographic, disease, or treatment factors and the clinical endpoints were tested using Chi-squared or Log Rank tests. All statistical tests were 2-sided, controlling for a probability of Type I error of 0.05. Statistical analyses were performed using Tibco Spotfire S + version 8.2 and SAS software version 9.4.

## Results

### Patient Characteristics

Eighteen patients were enrolled in this study. Demographics, baseline characteristics, and tumor types for these 18 patients are summarized in Table 1. The median age was 55 years (range, 25–75 years). Thirteen patients (72%) had GBM (of these, 6 were with methylated, 6 unmethylated, and 1 with unknown MGMT promoter methylation status); 2 patients had astrocytoma, IDH-mutant, grade 3 (one with methylated and one with unknown MGMT promoter status), and 3 patients had astrocytoma, IDH-mutant, grade 4 (2 with methylated and one with unmethylated MGMT promoter status). As per eligibility criteria, none of the patients had progressed previously while on TMZ. Classification of tumors was based on the 2021 World-Health Classification of tumors of the central nervous system.^18^

**Table 1.** Patient Characteristics. Diagnosis Per 2021 World Health Organization Classification of Tumors of the Central Nervous System

*Age*	
Mean (SD)	51.3 (15.0)
Median (range)	54.5 (25, 75)
*Sex (N, %)*	
Female	3 (17)
Male	15 (83)
*Race (N, %)*	
Non-Hispanic White	16 (89)
Black or African American	1 (6)
Hispanic White	1 (6)
*Diagnosis (N, %)*	
Glioblastoma, IDH-wild type	13 (72)
Astrocytoma, IDH-mutant, grade 4	3 (17)
Astrocytoma, IDH-mutant, grade 3	2 (11)
*Prior treatment*	
Radiation and temozolomide	18 (100)
*Death (N, %)*	
Expired	8 (44)
Alive	10 (56)
*Study site (N, %)*	
HPR	4 (22)
JHU	3 (17)
UIC	11 (61)
*Dose level (N, %)*	
1	7 (39)
2	8 (44)
3	3 (17)
*MGMT status (N, %)*	
Methylated	9 (50)
Non-Methylated	7 (39)
Unknown	2 (11)
*IDH mutation (N, %)*	
Negative	13 (72)
Positive	5 (28)

### Dose Escalation and MTD Assessment

Dose expansion was required for DLs 1 and 2. DL3, PAC-1 625 mg, and TMZ orally 150 mg/m^2^, were the highest studied and acceptable DL in this study. The study was discontinued due to lack of sufficient funding before patients could be enrolled in DL4. Hence, a MTD or RP2D could not be defined for the combination of PAC-1 and TMZ for patients with recurrent high-grade astrocytoma.

### Safety and Adverse Events

All 18 patients were included in the safety analysis. Drug-related adverse events did not result in treatment discontinuations. The incidence of grade 3 adverse events related to drugs occurred in 11% (2/18) of patients. These were alanine aminotransferase (ALT) and aspartate aminotransferase (AST) elevation in one patient at DL1, and lymphopenia in one patient at DL2. There were no recorded grade 4 toxicities. Table 2 shows all treatment-related events reported in the study.

**Table 2.** Adverse Events

Adverse Event	Grade 1	Grade 2	Grade 3	Grade 4
Dose Level 1: PAC-1 375 mg + TMZ 150 mg/m^2^				
* Hematologic*				
Anemia	3	0	0	0
Leukopenia	1	1	0	0
Lymphopenia	1	2	0	0
* Non-Hematologic*				
Fatigue	3	2	0	0
Fever	1	0	0	0
Confusion	1	0	0	0
Somnolence	0	1	0	0
Neurologic, other	3	0	0	0
Concentration impairment	1	0	0	0
Dysgeusia	1	0	0	0
Bruising	1	0	0	0
Arthralgia	1	0	0	0
ALT increase	0	0	1	0
AST increase				
Dose Level 2: PAC-1 500 mg + TMZ 150 mg/m^2^				
* Non-Hematologic*				
Cognitive disturbance	0	1	0	0
Headache	0	1	0	0
Paresthesia	0	1	0	0
Neurologic, other	1	0	0	0
Dose Level 3: PAC-1 625 mg + TMZ 150 mg/m^2^				
* Non-Hematologic*				
Fatigue	2	0	0	0
Dizziness	1	0	0	0
Headache	1	0	0	0
Constipation	1	0	0	0
Diarrhea	1	0	0	0

### Efficacy Data

**Figure 1.**
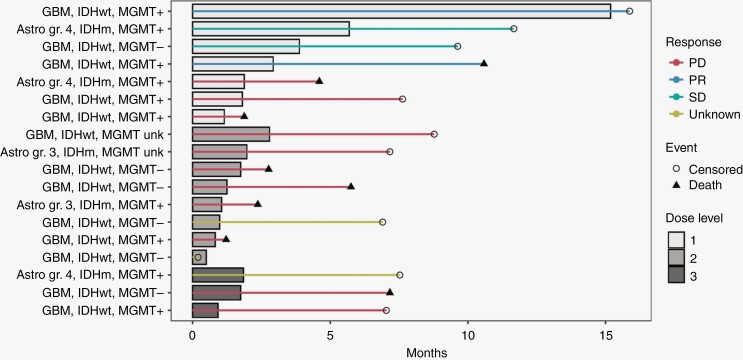
Swimmer plot illustrating diagnosis of each study participant, IDH-mutation and MGMT promoter methylation status, response, time on trial and in follow-up, sorted by PAC-1 dose level. Astro = astrocytoma.

Two patients (11%) had a confirmed partial response with 73 and 46% reduction in cross-diametrical product of the contrast-enhancing target lesion, including one patient that remained on therapy for 15 months. These were both at DL1, both with GBM, IDH-wild type, MGMT promoter methylated. There were 2 patients with stable disease (11%), 11 patients with progressive disease (61%), and 3 patients without sufficient available data to assess response (17%). By the end of data collection, 10 patients (56%) were alive (median time to last follow-up, 7.6 months), and 8 had died (44%). Figure 1 illustrates response and treatment data for all 18 patients in this study.

### Pharmacokinetics

**Figure 2.**
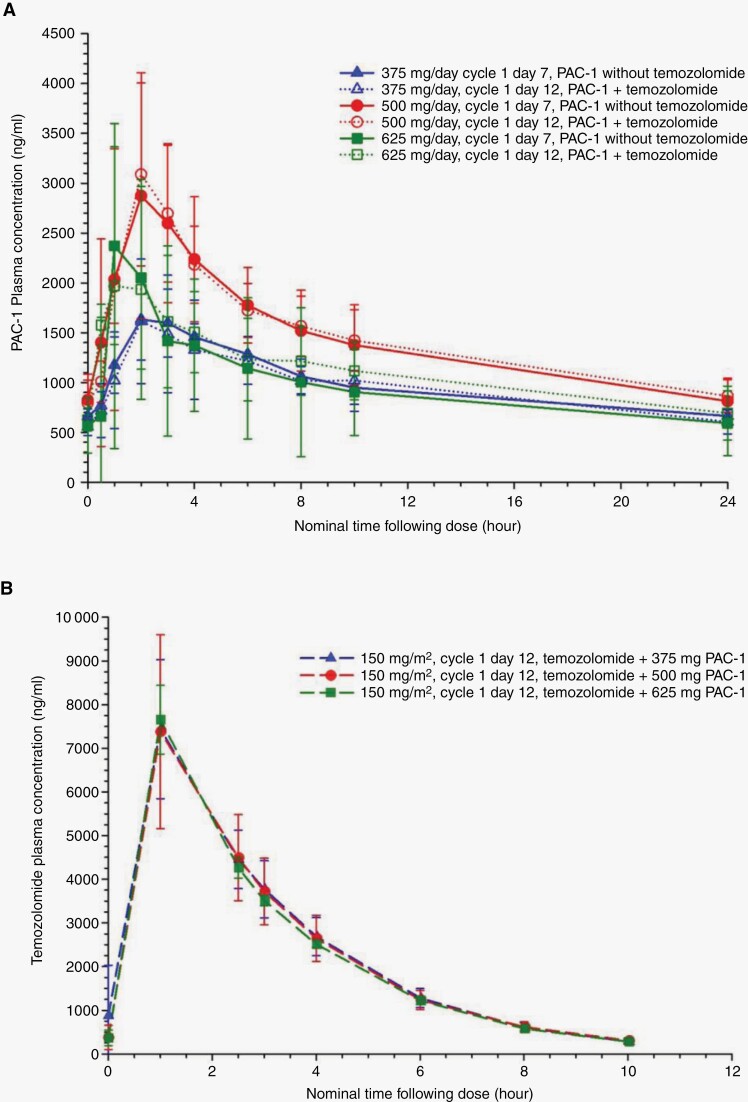
(A) Mean (± standard deviation) PAC-1 plasma concentration-time profiles at steady-state following multiple oral doses of 375, 500, or 625 mg once daily without temozolomide (cycle 1, day 7) or with temozolomide (cycle 1, day 12). (B) Mean (± standard deviation) temozolomide plasma concentration-time profiles following multiple oral doses of 150 mg/m^2^ administered concurrently with 375, 500, or 625 mg of oral PAC-1 once daily.

Figure 2A and B display the mean PAC-1 and TMZ plasma concentration versus time curves among the 3 dose groups and, for PAC-1, administration without and with TMZ. Within each dose group, plasma concentrations of PAC-1 were similar when administered in the absence or presence of TMZ. The PAC-1 plasma concentrations increased proportionally between 375 mg/day and 500 mg/day. However, mean PAC-1 plasma concentrations at 625 mg/day unexpectedly fell below the mean concentrations at 500 mg/day. This incongruity reflected a single subject in the 625 mg group, whose PAC-1 plasma concentrations were approximately 70% below predicted levels. The reduction in PAC-1 concentrations were the same without and with concurrent TMZ. The PAC-1 plasma concentrations for the other 2 subjects in the 625 mg group were also lower than expected. However, the decrement in concentrations in these subjects was smaller, 10% to 30% below predicted mean concentrations, and fell within the inter-subject variability of PAC-1 PK.^14^

The pharmacokinetic parameters for PAC-1 are summarized in Supplementary Table 1. The only parameter showing a statistically significant difference (*P* < .05) during concurrent administration of TMZ was C_min,ss_. The 90% CIs for the geometric mean ratios of PAC-1 C_max_, AUC_0--_,_ss_, CL/F, and t_1/2_ with and without concurrent administration of TMZ were contained within the bioequivalence levels of 0.80 and 1.25 (Supplementary Table 1). The geometric mean ratio for PAC-1 V/F in the presence and absence of TMZ was 0.99. However, a lack of effect of TMZ on PAC-1 V/F could not be concluded because the lower 90% CI of 0.77 fell slightly below 0.80.

The mean plasma concentrations of TMZ were nearly identical across the 3 PAC-1 dose groups (Figure 2B). The mixed effects analyses found that PAC-1 dose did not significantly (p > 0.05) impact any PK parameters of TMZ (Supplementary Table 2). The 90% CI for the geometric mean ratios between PAC-1 dose groups for AUC _0-∞_ fit within the bioequivalence limits. For C_max_, CL/F, V/F, and t_1/2_, the 90% CIs for one or more of the between-dose group comparisons were outside the 0.80 to 1.25 acceptability criteria. The differences between the errant CIs and bioequivalence levels were modest, with the smallest lower 90% CI of 0.75 and largest upper 90% CI of 1.28.

### NCF Data

Sixteen patients completed NCF testing at one or more time points: *n* = 7 patients at DL1, *n* = 6 at DL2, and *n* = 3 at DL3. At baseline, patients entered the study with widespread NCF impairments consistent with their disease and treatment history (see Supplementary Table 3). When examining changes in standardized scores over time, the CTB COMP did not suggest trends of global neurocognitive dysfunction over time (see Supplementary Figure 3). No clear adverse effect of dose or time on the study drug was seen on any of the NCF tests at the group level; average performance on NCF tests over time were generally better for the highest dose group compared to the lower 2 dose groups.

### Immunohistochemistry

Six archival tumor tissues underwent additional histologic evaluation. Five of the six tumors demonstrated cytoplasmic overexpression of PC-3 and staining intensities were graded as strong (2/6), moderate (2/6), faint (1/6), and negative (1/6) (Supplementary Figure 1). In one GBM tumor graded as negative for cytoplastic PC-3, distinct nucleolar staining for PC-3 was noted and of undetermined significance. Low levels of CC3-positive malignant glial cells were identified in all samples, supporting ongoing apoptotic cell death within the tumor population at the time of tumor biopsy or resection. In some specimens, islands of cellular necrosis were identified and associated with previous radiation and chemotherapeutic interventions administered temporally close to the time of tissue biopsy and collection. Nuclear immunostaining for MGMT was identified in 4 tumor samples. Immunohistochemical detection of MGMT tracked accordingly with known methylated or unmethylated MGMT promoter status assessed from concurrently collected fresh frozen tissue specimens in 3 of the 4 tumor samples.

## Discussion

In this study, we demonstrated that the combination of PAC-1 and TMZ was safe and well tolerated to the highest dose studied. Due to limited funding, dose escalation was stopped at PAC-1 625 mg orally daily and TMZ 150 mg/m^2^/5 days per 28-day cycle.

Two patients had a partial response to PAC-1 and TMZ at DL1; both with GBM, IDH-wild type and both with methylated MGMT promoter. It cannot be determined if the responses were due to TMZ alone and whether PAC-1 contributed to the clinical benefit or not.

We did not identify PK interactions between PAC-1 and TMZ. The PK parameters observed in our study, including the range of values and interindividual variability, agree with other reports of PAC-1^14^ and TMZ in cancer patients.^19-21^Co-administration of oral PAC-1 at doses of 375, 500, or 625 mg/day with TMZ at a daily dose of 150 mg/m^2^ had minimal impact on the PK of TMZ. The 90% confidence interval for the geometric mean ratios confirmed that TMZ did not significantly affect the peak and overall exposure of PAC-1. A nonsignificant effect of PAC-1 on TMZ PK was supported by the similarity between PK parameters in the present report and values reported in the literature in patients not receiving PAC-1.^19–21^ The less than proportional change in PAC-1 exposure between the 625 mg group and 375 mg and 500 mg groups was unanticipated; potential explanations for this are discussed in detail in Supplementary Material.

Our study had several limitations. These include the relatively small samples size and the heterogeneity of patients. We included both patients with astrocytomas grade 3 and 4 because the primary endpoint of this study was safety and tolerability; however, this certainly complicates interpretation of efficacy as IDH-mutant and/or MGMT promoter methylated gliomas may well respond to TMZ alone. In addition, we only studied one dose level of TMZ, 150 mg/m2 for 5 days per cycle. The rationale for this was concern for myelotoxicity and other TMZ-related side effects with the higher dose of TMZ 200 mg/m^2^, and to allow more cycles in responders.

Based on the encouraging preclinical data with PAC-1 in gliomas, including in vivo evidence of crossing the blood-brain barrier in mice, its observed safety and tolerability in this study, as well as its pharmacokinetic properties in combination with TMZ, we feel that this drug deserves further investigation in the treatment of gliomas. Further dose escalation, to determine the MTD and RP2D, will be necessary for the combination of PAC-1 and TMZ prior to evaluating this drug combination prospectively for efficacy. For formal efficacy testing, a homogeneous patient cohort should be chosen, considering MGMT promoter methylation and IDH-mutation status, so that efficacy, or lack thereof, can be clearly defined. Another possible direction to further study PAC-1 in MGMT promoter unmethylated GBM would be to evaluate activity and safety of PAC-1 and radiation (based on previously reported activity of this combined treatment modality in canine patients^8^), or together with TMZ in MGMT promoter methylated GBM.

## Supplementary Material

vdad087_suppl_Supplementary_Materials
