# Supplementary material for: Phase I dose-escalation study of procaspase-activating compound-1 in combination with temozolomide in patients with recurrent high-grade astrocytomas
Source: Neurooncol Adv. 2023 Jul 19;5(1):vdad087. doi: 10.1093/noajnl/vdad087 (PMC10406430; doi:10.1093/noajnl/vdad087)
Supplement: vdad087_suppl_Supplementary_Materials [file vdad087_suppl_supplementary_materials.zip › vdad087_suppl_supplementary_materials_edited_mh_7.21.23.docx]

**SupplementaRY Material**

1. **Study Procedures**

***Definition of a DLT***

A DLT was defined as one of the following treatment-related events occurring during the first cycle with exception of neurological toxicity: a) grade 3 or greater treatment-related hematologic toxicity for > 48 hours during the first cycle (28 days) of therapy; b) cerebrovascular ischemia or hemorrhage of any duration or grade; c) grade 3 or greater treatment-related clinical non-hematological toxicity (excluding ≥ grade 3 nausea, vomiting, or diarrhea without maximal medical intervention and/or prophylaxis) during the first cycle (28 days) of therapy; d) delay of cycle 2 treatment start by more than 2 weeks due to incomplete hematologic recovery (ANC > 1.5 x 10^9^/L or platelets > 100 x 10^9^/L) or unresolved treatment related grade 3 or greater non-hematologic toxicity; e) grade 2 or greater treatment-related neurological toxicity occurring during the first 2 cycles of therapy and lasting more than 72 hours.

1. **Pharmacokinetic Analysis**

***Statistical Considerations***

The PAC-1 and temozolomide PK parameters were summarized as the geometric mean and coefficient of variation, except T_max_ that was expressed as the median and range. The influence of temozolomide on PAC-1 PK was evaluated using a mixed-effects generalized linear models approach with presence of temozolomide, dose group, and interaction term as fixed effects and subject within temozolomide treatment as a random effect. The PK parameters were logarithmically (natural) transformed and C_ss-max_, C_ss-min_, and AUC_0-τ_,_ss_ normalized for a 500 mg dose across dose groups for the analysis by multiplying the parameter by the quotient of 500 mg divided by the actual dose. The geometric least square mean ratio, PAC-1 with temozolomide compared to PAC-1 without temozolomide and 90% confidence interval (CI) for each parameter, were constructed from the linear mixed effects analysis. Lack of interaction was concluded when the 90% CI for the ratio was contained within the conventional bioequivalence range of 0.80 to 1.25. Although no data were obtained from administration of temozolomide without PAC-1, the influence of PAC-1 dose on temozolomide PK was evaluated using a similar mixed effects approach.

The less than proportional change in PAC-1 exposure between the 625 mg group and 375 mg and 500 mg groups was unanticipated; as a phase 1 trial evaluating PAC-1 as a single agent in treating solid tumors observed a linear relationship between PAC-1 dose and AUC at doses up to 750 mg/day.^13^ Non-compliance with their PAC-1 regimen represents a possible cause for the unexpected observations among the subjects in the 625 mg dose group. However, analysis of drug administration records and the similar PAC-1 plasma concentrations at both PK evaluations lessen the probability of non-compliance.

Alternatively, a PK mechanism may explain the lower than predicted PAC-1 exposure at 625 mg/day. The discordant PAC-1 plasma concentrations at 625 mg/day signify a higher PAC-1 CL/F compared to the 375 mg/day and 500 mg/day dose groups. Data from animal studies and a single clinical trial imply that PAC-1 is a high extraction ratio, hepatically eliminated drug with low and variable oral bioavailability.^1-3^ Preliminary evidence from *in vitro* studies point to cytochrome P450 isozymes (CYP) 2C19, 2D6, and 3A4 as possible pathways of hepatic metabolism (unpublished). Based on these properties, a reduction in absorption from the gastrointestinal tract, decrease in plasma protein binding, or increase in hepatic metabolism in the 625 mg group compared to the 375 mg and 500 mg groups would produce an increased CL/F.^4^ Review of demographics, laboratory results, and concurrent drugs and medical conditions did not identify differences among the 3 dose groups likely to impact gastrointestinal absorption or plasma protein binding of PAC-1. A relative increase in hepatic metabolism in the 625 mg group may result from either subjects in the 625 mg/day group receiving a drug that induces CYP2C19, 2D6, or 3A4 or subjects in the 375 and 500 mg/day groups receiving a drug that inhibits CYP2C19, 2D6, or 3A4. The only concomitant medication meeting these criteria was dexamethasone, a weak inducer of CYP3A4.^5^ Dexamethasone was co-administered with PAC-1 to all three subjects in the 625 mg/day group compared to 2 of 7 subjects in the 375 mg/day group and 5 of 8 subjects in the 500 mg/day group. Comparison of the PAC-1 CL/F between subjects from the 375 and 500 mg/day groups receiving and not receiving dexamethasone, 221 ml/h/kg versus 188 mg/h/kg, also suggest induction of PAC-1 CL/F by dexamethasone. However, several factors raise uncertainty concerning the contribution of dexamethasone to the lower than predicted PAC-1 plasma concentrations in the 650 mg/day dose group, including its relatively modest impact on CYP3A4 activity,^5^ the small increase in PAC-1 CL/F (approximately 18%) observed with dexamethasone co-administration in the 375 and 500 mg/day groups, and limited information on the pathways mediating PAC-1 metabolism. Further study is required to understand the cause and relevance of the less than proportional increase in PAC-1 exposure at 625 mg/day.

References:

1. Danciu OC, Holdhoff M, Peterson RA, et al. Phase I study of procaspase-activating compound-1 (PAC-1) in the treatment of advanced malignancies. *Br J Cancer.* 2023;128(5):783-792.
2. Ren L, Bi K, Gong P, et al. Characterization of the *in vivo* and *in vitro* metabolic profile of PAC-1 using liquid chromatography-mass spectrometry. *J Chromatogr B.* 2008;876(1):47-53.
3. Lucas PW, Schmit JM, Peterson QP, et al. Pharmacokientics and derivation of an anticancer dosing regimen for PAC-1 in healthy dogs. *Invest New Drugs.* 2011;29(5):901-911.
4. Wilkinson GR, Shand DG. Commentary: a physiological approach to hepatic drug clearance. *Clin Pharmacol Ther.* 1975;18(4):377-90.
5. Al Rihani SB, Deodhar M, Dow P, Turgeon J, Michaud V. Is Dexamethasone a Substrate, an Inducer, or a Substrate-Inducer of CYP3As?. *Arch Phar Pharmacol Res.* 2020 ; 2(4): 1-5.

**Supplementary Table 1**: Summary of pharmacokinetic parameters for PAC-1 following 375 mg, 500 mg, or 625 mg administered once daily orally alone (Cycle 1 Day 7) and concurrently with temozolomide (Cycle 1 Day 12).

| **Parameter** | **Geometric Mean (geometric CV%)** | | | | | | **Geometric Least-Square Mean Ratio (90%CI)**  **Cycle 1 Day 12/ Cycle 1 Day 7** |
| --- | --- | --- | --- | --- | --- | --- | --- |
|  | **375 mg Dose Group** | | **500mg Dose Group** | | **625 mg Dose Group** | |  |
|  | **Cycle 1 Day 7** | **Cycle 1 Day 12** | **Cycle 1 Day 7** | **Cycle 1 Day 12** | **Cycle 1 Day 7** | **Cycle 1 Day 12** |  |
| Number of subjects | 7 | 6 | 8 | 6 | 3 | 3 | ----- |
| T_max_ (h)^*^ | 3.00 (1.98-6.00) | 3.00 (1.98 - 9.83) | 2.34 (0.50- 5.92) | 2.01 (1.92 - 3.07) | 1.98 (0.82 – 2.00) | 1.03 (1.00 - 4.17) | ----- |
| C_max_ (ng/ml) | 1728 (19.6) | 1802 (19.7) | 3026 (34.0) | 2938 (32.2) | 1876 (47.5) | 2124 (57.9) | 1.02 (0.85, 1.23) ^†^ |
| C_ss-min_ (ng/ml) | 647.8 (11.0) | 525.4 (12.3) | 776.8 (27.9) | 770.0 (19.6) | 565.9 (47.4) | 480.0 (40.4) | 0.86 (0.80, 0.92) ^†^ |
| AUC_ss,0-τ_ (h*ng/ml) | 23434 (12.0) | 22519 (13.8) | 32823 (28.0) | 34274 (13.2) | 24634 (45.2) | 21854 (39.7) | 0.93 (0.85, 1.02) ^†^ |
| CL/F (ml/h/kg) | 185 (26.6) | 193 (32.9) | 203 (38.1) | 194 (26.0) | 322 (38.0) | 363 (31.2) | 1.07 (0.98, 1.17) |
| V/F (L/kg) | 6.1 (36.1) | 5.4 (27.3) | 5.1 (53.0) | 5.0 (40.3) | 8.7 (40.6) | 9.7 (17.0) | 0.99 (0.77, 1.26) |
| t_1/2-λ_ (h) | 22.9 ((24.6) | 20.0 (12.0) | 17.6 (18.9) | 17.9 (31.1) | 18.7 (42.9) | 18.5 (32.9) | 1.04 (0.90, 1.22) |

* T_max_ – summarized a median (range), ^†^ Values normalized to 500 mg for calculation of geometric ratio, CV%- coefficient of variation expressed as percent, 90% CI- 90% confidence interval, AUC_0-τ,_ss -area under the PAC-1 plasma concentration-time curve over 24 hour dosing interval at steady-state, C_max_ - maximum PAC-1 plasma concentration at steady-state, T_max_ - time to achieve C_max_, C_ss-min_ - minimum PAC-1 plasma concentration at steady-state, CL/F- oral clearance, V/F- apparent volume of distribution, t_1/2-λ_ - terminal elimination half-life.

**Supplementary Table 2**: Summary of pharmacokinetic parameters for temozolomide following 150 mg/m^2^ administered orally once daily with oral PAC-1 375 mg, 500 mg, or 625 mg per day.

| **Parameter** | **Geometric Mean (geometric CV%)** | | | **Geometric Least-Square Mean Ratio (90%CI)** | | |
| --- | --- | --- | --- | --- | --- | --- |
|  | **375 mg Dose Group** | **500mg Dose Group** | **625 mg Dose Group** | **375 mg/500 mg Dose Groups** | **375 mg/625 mg Dose Groups** | **500 mg/625 mg Dose Groups** |
| Number of subjects | 6 | 7 | 3 | --- | --- | --- |
| T_max_ (h)^*^ | 1.00 (0.82 - 2.35) | 0.98 (0.82 – 2.50) | 1.00 (0.82 – 1.00) | --- | --- | --- |
| C_max_ (ng/ml) | 7306 (22.1) | 7748 (18.0) | 7626 (10.3) | 94 (0.78,1.13) | 0.96 (0.76,1.21) | 1.02 (0.81,1.28) |
| AUC_0-∞_ (h*ng/ml) | 22774 (7.4) | 23755 (10.5) | 23896 (9.5) | 0.96 (0.87,1.05) | 0.95 (0.84,1.07) | 0.99 (0.88,1.12) |
| CL/F (ml/h/kg) | 151 (17.2) | 158 (13.3) | 160 (22.7) | 0.95 (0.81,1.12) | 0.94 (0.77,1.15) | 0.98 (0.81,1.20) |
| V /F (L/kg) | 0.40 (16.7) | 0.44 (15.0) | 0.44 (15.7) | 0.91 (0.78,1.08) | 0.91 (0.75,1.12) | 0.99 (0.82,1.21) |
| t_1/2-λ_ (h) | 1.86 (5.3) | 1.93 (10.1) | 1.92 (7.0) | 0.96 (0.89,1.04) | 0.97 (0.88,1.07) | 1.01 (0.81,1.28) |

* T_max_ – summarized a median (range), CV%- coefficient of variation expressed as percent, 90% CI- 90% confidence interval, AUC_0-∞_ -area under the temozolomide plasma concentration-time curve from time zero to infinity, C_max_ - maximum temozolomide plasma concentration, T_max_ - time to achieve C_max_, CL/F- oral clearance, V /F- apparent volume of distribution, t_1/2-λ_ - terminal elimination half-life.

1. **Immunohistochemistry**

***Immunohistochemistry***

Immunohistochemical (IHC) staining was performed on formalin-fixed, paraffin-embedded (FFPE) tumor tissues using an indirect immunoperoxidase technique with diaminobenzidine (DAB) as the chromogen for single-plex analysis of PC-3, cleaved caspase-3 (CC3), or MGMT and IHC staining was performed using an autostainer (intelliPATH FLX, Biocare, Concord, CA). Processed slides were deparaffinized in xylene and rehydrated in alcohol. Endogenous peroxidase activity was blocked with Biocare PX968 Peroxidazed 1 at RT for 5 minutes, rinsed with TBS wash buffer, and then incubated for 10 minutes at RT with Biocare BP974 Background Punisher. Slides were incubated with PC-3 antibody (1:3000; Abcam, ab32150) for 30 minutes, CC3 antibody (1:100; Cell Signaling, #9661) at 4°C overnight, or MGMT antibody (1:2000; Abcam, ab108630) for 30 minutes. Following primary antibody incubation, all IHC single-plex slides were washed, and then incubated with HRP-Polymer (Biocare, RC542) for 30 minutes. Slides were washed with TBS, then the reaction was developed using DAB substrate for 5 minutes. Slides were counterstained with Mayer’s hematoxylin. For PC-3 or CC3 immunostainings, human lymph node or tonsil served as an internal positive control, respectively. For MGMT immunostaining, MCF7 or U87MG cell pellets were formalin fixed, embedded in agarose, and processed identically to archived tissue samples, and used as positive or negative controls, respectively. Scoring of immunoreactivity data is summarized in below.

***Scoring of immunoreactivity data***

Six archival tumor tissues from 6 patients presenting for localized adult-type diffuse glioma recurrence at a single institute (UIC) were available for additional histologic evaluation. All 6 tumors were evaluated for PC-3, CC3, and MGMT immunohistochemical staining. The use of human tumor samples in the research conducted was approved by the Human Subjects Institutional Review Board at three participating institutes, the University of Illinois at Chicago (Chicago, Illinois, USA), Johns Hopkins Kimmel Cancer Center (Baltimore, Maryland, USA) and Regions Hospital (Saint Paul, Minnesota, USA). Samples were de-identified and assigned a 5-digit numerical designation.

For PC-3 immunoreactivity, up to 500 cells—or as many as were available—from each sample were graded by one observer (TMF), and the percentage of negative, faintly staining, moderately staining, and strongly staining cells were recorded. Negatively staining samples contained <10% PC-3 positive cells. Cells that had <50% cytoplasmic staining were graded as “faintly stained,” those with >50% cytoplasmic staining were graded as “moderately stained,” and those with >50% cytoplasmic staining and in which nuclear detail was obscured by staining intensity were categorized as “strongly stained.”

**Supplementary Figure 1:**

Histologic (hematoxylin and eosin) and immunohistochemical (PC-3, CC3, and MGMT) evaluation of recurrent malignant glial tumors demonstrating opportunities to target potential enzymatic vulnerabilities. Overexpression of cytoplastic PC-3 and absence of nuclear MGMT in recurrent adult-type diffuse glioma support the rational combination of PAC-1 and temozolomide in a subset of patients diagnosed with recurrent malignant glial tumors. Magnification 400x, scale bar 50 microns.

*
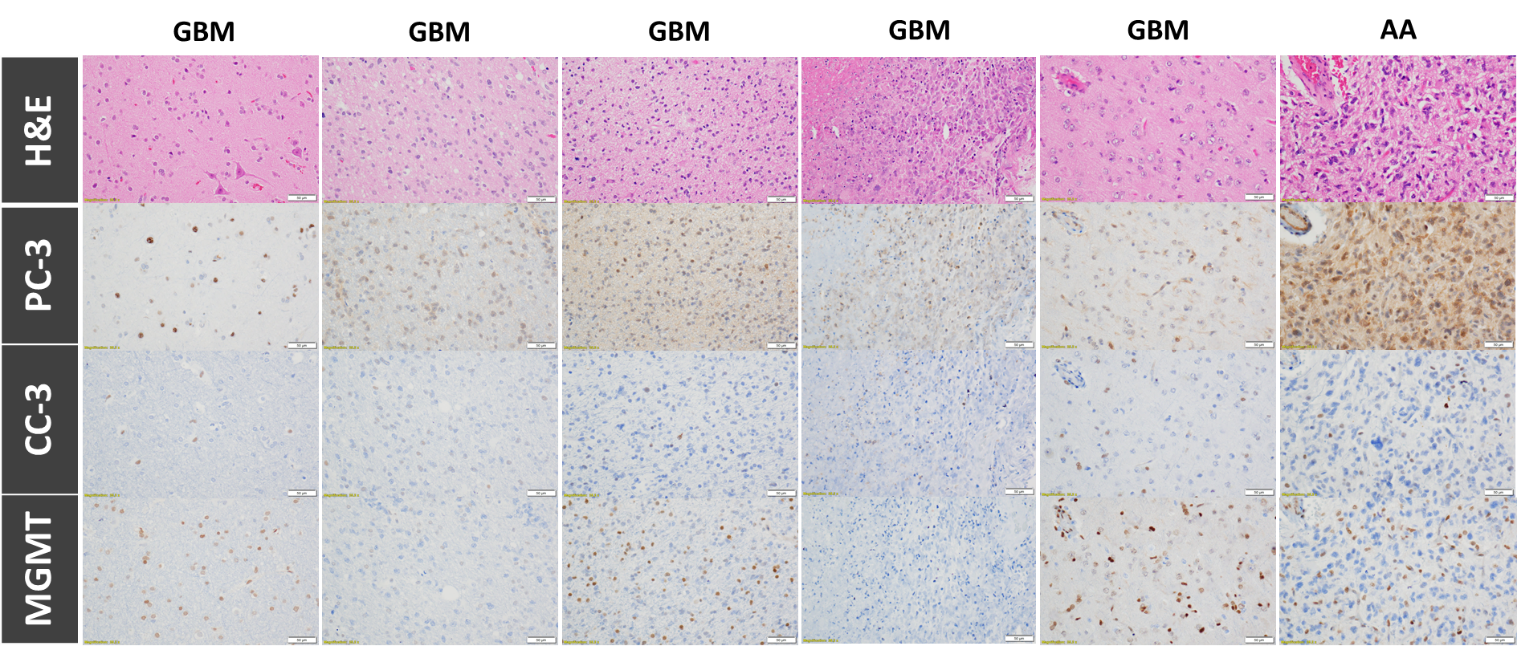
*

**GBM**

**AA**

**GBM**

**GBM**

IV. **Neurocognitive Function**

***Training and Certification***

All potential test administrators seeking certification and approval to administer the NCF tests to patients on study were required to read the protocol, read the study-specific neurocognitive test administration and training manual, watch a neurocognitive training video, complete a training post-test assessing knowledge about standardized test administration, and demonstrate accurate test administration. All training materials and activities were reviewed by Dr. Jeffrey Wefel. Any procedural errors were reviewed with the trainee and additional training was provided as required until familiarity with the tests was confirmed and the individual was approved to administer the tests to study patients.

***NCF Test Administration***

The NCF test battery was administered according to the assessment schedule in the protocol and prior to patients receiving information about the status of their disease. NCF testing must be completed in one session in a quiet, distraction free environment. All NCF test forms were submitted to Dr. Wefel for blinded, centralized scoring.

***NCF Statistical Methods***

For each test at each time point, the tests’ raw scores were converted to demographic-adjusted z-scores based upon published normative data from healthy populations. The Clinical Trial Battery Composite (CTB COMP) score is calculated as the arithmetic mean of the standardized scores for each test divided by the total number of tests (minimally 5 tests must be included to calculate the CTB COMP).

***Change in standardized scores over time***

A change in standardized score (z-score) for each test at each follow-up time point relative to baseline was calculated for each dose level and each patient clustered by dose level. Change scores were plotted over time by treatment arm/dose level.

***Neurocognitive Adverse Event***

Change in standardized test scores from baseline to each follow up time point in z-score units was calculated (follow up – baseline), such that any negative number reflects a test performance at follow-up that is worse than at baseline. In the interest of mapping changes in neurocognitive test scores onto the framework of the National Cancer Institute Common Terminology Criteria for Adverse Events (NCI CTCAE), Version 4.0, an ad hoc definition of Grade 1-3 neurocognitive adverse events (NCAE) was defined based on changes in z-score units. Importantly, the designation of “adverse event” should not be inferred to mean that this is necessarily attributable to study drug.

**Ad Hoc Definition of Grade 1-3 Neurocognitive Adverse Effects**

| **NCAE Severity** | **Qualitative Label** | **Quantitative z-Score Change** |
| --- | --- | --- |
| Grade 1 | Mild | -1.00 to -1.99 |
| Grade 2 | Moderate | -2.00 to -2.99 |
| Grade 3 | Severe | >/= -3.00 or new inability to complete a test that the patient was previously able to complete |

***Reliable Change Index (RCI)-defined Decline***

Change in raw scores from baseline to each follow-up time point was calculated (follow-up – baseline). Negative numbers reflect a test performance at follow-up that is worse than at baseline for the HVLT-R and COWA. Positive numbers reflect a test performance at follow-up that is worse than at baseline for the TMT. Based on the RCI value for each test, change in test scores from baseline to each follow-up time point were determined to represent either RCI-defined Decline or No Decline. Additionally, any new inability to complete a test that the patient was previously able to complete was to be considered a Decline. Importantly, the designation of “RCI-defined Decline” was not to be inferred to mean that this was necessarily attributable to study drug.

**Supplementary Table 3 – Baseline Neurocognitive Test Performance by Dose Level**

| **Arm** | **HVLT-R TR** | **HVLT-R DR** | **HVLT-R RECOG** | **TMTA** | **TMTB** | **COWA** | **CTB COMP** |
| --- | --- | --- | --- | --- | --- | --- | --- |
| **All Dose Levels**  **M+/-SD**  **Min, Max** | -2.04 (1.58)  -4.77, 1.05 | -1.88 (2.14)  -6.06, 1.22 | -1.07 (1.85)  -4.86, 0.86 | -3.47 (11.79)  -44.16, 1.32 | -5.62 (9.80)  -39.18, 0.88 | -1.76 (0.80)  -3.21, -0.46 | -2.50 (3.57)  -14.66, -0.37 |
| **Dose Level 1**  **M+/-SD**  **Min, Max** | -2.06 (2.18)  -4.77, 1.05 | -2.73 (2.80)  -6.06, 1.22 | -1.46 (2.48)  -4.86, 0.86 | -9.54 (19.42)  -44.16, 1.32 | -9.96 (14.48)  -39.18, -1.72 | -2.11 (0.76)  -3.21, -0.97 | -4.18 (5.39)  -14.66, -0.37 |
| **Dose Level 2**  **M+/-SD**  **Min, Max** | -2.04 (1.31)  -3.89, -0.21 | -1.63 (1.24)  -3.71, -0.44 | -1.02 (1.37)  -2.91, 0.86 | -0.36 (1.50)  -3.14, 1.09 | -3.01 (3.82)  -10.07, 0.88 | -1.07 (0.52)  -1.89, -0.46 | -1.52 (1.00)  -2.87, -0.58 |
| **Dose Level 3**  **M+/-SD**  **Min, Max** | -2.00 (0.47)  -2.44, -1.51 | -0.38 (1.01)  -1.35, 0.67 | -0.27 (1.01)  -1.09, 0.86 | 0.45 (0.50)  -0.13, 0.75 | -2.15 (4.02)  -6.75, 0.65 | -2.30 (0.37)  -2.60, -1.89 | -1.11 (0.30)  -1.40, -0.80 |

**Supplementary Figure 2 - Mean Z-Score Change from Baseline for all Tests by Dose Level over Time.**


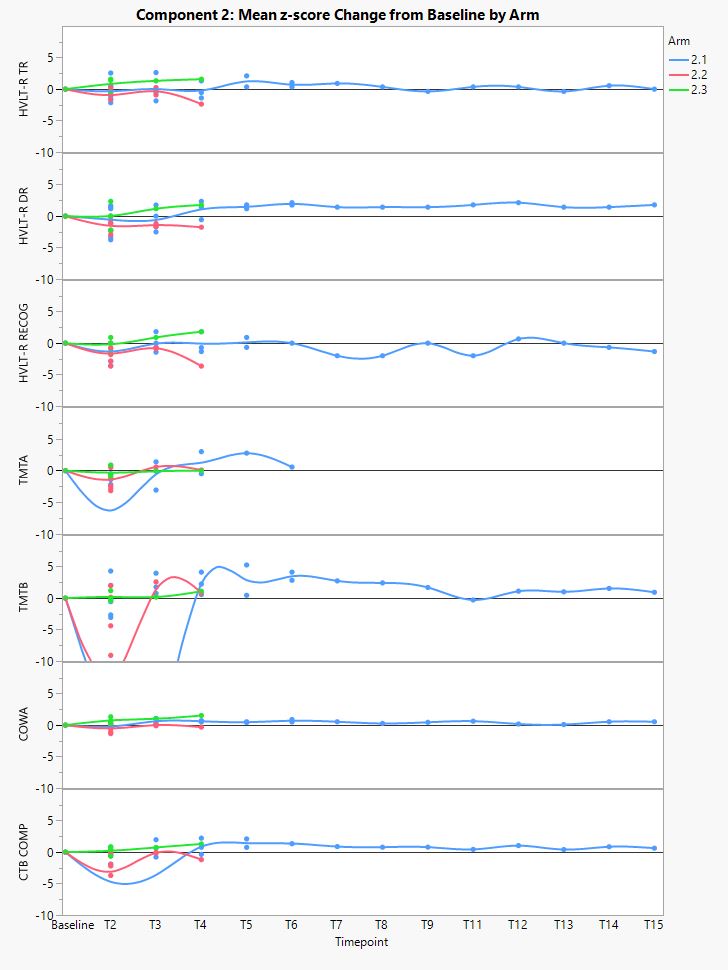


Note: Arm 2.1 = Dose Level 1, Arm 2.2 = Dose Level 2, Arm 2.3 = Dose Level 3

Changes in the continuous standardized neurocognitive test scores over time did not show a clear relationship between declines in NCF and dose of PAC-1 or time on PAC-1. At the mean score level of analysis, early declines were generally followed by subsequent improvement. Average performance on NCF tests over time were generally better for the highest dose group (dose level 3) compared to the lower two dose groups. The lowest dose level group showed a profound decline on TMTA and TMTB at time 2 (day 1, cycle 2). This was due to a single patient who was identified to have a clinically significant change on neurologic exam at that time point as well. Follow-up testing at time 3 (day 1, cycle 3) demonstrated a full recovery of performance on TMTA and a partial improvement at time 3 on TMTB. Mean performance in dose level 2 showed a profound decline on TMTB at time 2; four out of six patients evidenced decline at time 2. Only one patient completed testing at the 30-day follow-up and exhibited evidence of partial recovery.

***Neurocognitive Adverse Event (NCAE)***

The frequency of NCAEs per test by dose level while on treatment are summarized in Table 4. As can be seen in Table 4, NCAEs of any grade occurred at all dose levels but with fewer NCAEs at the highest dose level tested (dose level 3 = 10%) and the most NCAEs in dose level 2 (54%). Grade 3 NCAEs were more frequent in dose level 2 (17%) and least frequent in dose level 3 (0%). NCAEs of any grade were most common on the HVLT-R DR and RECOG (i.e., memory). Grade 3 NCAEs were most common on the HVLT-R RECOG (i.e., memory) and TMTB (i.e., executive function). Of the 19 Grade 3 NCAEs during treatment there was no further follow up testing to evaluate persistence or resolution of the NCAE in 8 cases. In the other 11 cases, 9 improved in the severity of the NCAE on follow up testing and 2 exhibited a persistent Grade 3 NCAE.

**Supplementary Table 4. Frequency and Severity of Neurocognitive Adverse Events (NCAE) by Dose Level While on Treatment.**

|  | **Arm** | **n** | **Patients with NCAE Event**  **n (%)** | **Total NCAE**  **Events**  **n** |  | **Proportion of Visits with NCAE Event**  **n (%)** | | **Grade 1**  **n** | **Grade 2**  **n** | | **Grade 3**  **n** | | **Proportion of Visits with Any and with Grade 3 NCAE**  **n (%)** | |  |
| --- | --- | --- | --- | --- | --- | --- | --- | --- | --- | --- | --- | --- | --- | --- | --- |
| **HVLT-R TR** | DL1 | 7 | 3 (43%) | 5 | 5/26 | | (19%) | 4 | | 1 | | 0 | Any: 30/141  Gr3: 10/141 | (21%)  (7%) | |
| **HVLT-R DR** | DL1 | 7 | 3 (43%) | 5 | 5/26 | | (19%) | 2 | | 1 | | 2 |  |  |  |
| **HVLT-R RECOG** | DL1 | 7 | 4 (57%) | 9 | 9/26 | | (35%) | 3 | | 5 | | 1 |  |  |  |
| **TMTA** | DL1 | 5 | 4 (80%) | 4 | 4/12 | | (33%) | 1 | | 1 | | 2 |  |  |  |
| **TMTB** | DL1 | 6 | 3 (50%) | 4 | 4/25 | | (16%) | 0 | | 1 | | 3 |  |  |  |
| **COWA** | DL1 | 7 | 0 (0 %) | 0 | 0/26 | | (0%) | 0 | | 0 | | 0 |  |  |  |
| **CTB COMP** | DL1 | 6 | 2 (33%) | 3 | 3/25 | | (12%) | 1 | | 0 | | 2 |  |  |  |
| **HVLT-R TR** | DL2 | 6 | 4 (67%) | 4 | 4/9 | | (44%) | 3 | | 1 | | 0 | Any: 29/54  Gr3: 9/54 | (54%)  (17%) | |
| **HVLT-R DR** | DL2 | 6 | 5 (83%) | 7 | 7/9 | | (78%) | 4 | | 3 | | 0 |  |  |  |
| **HVLT-R RECOG** | DL2 | 6 | 4 (67%) | 4 | 4/9 | | (44%) | 1 | | 1 | | 2 |  |  |  |
| **TMTA** | DL2 | 6 | 4 (67%) | 4 | 4/9 | | (44%) | 1 | | 2 | | 1 |  |  |  |
| **TMTB** | DL2 | 6 | 4 (67%) | 4 | 4/9 | | (44%) | 0 | | 0 | | 4 |  |  |  |
| **COWA** | DL2 | 6 | 2 (33%) | 2 | 2/9 | | (22%) | 2 | | 0 | | 0 |  |  |  |
| **CTB COMP** | DL2 | 6 | 5 (83%) | 5 | 5/9 | | (56%) | 2 | | 1 | | 2 |  |  |  |
| **HVLT-R TR** | DL3 | 3 | 0 (0%) | 0 | 0/5 | | (0%) | 0 | | 0 | | 0 | Any: 3/30  Gr3: 0/30 | (10%)  (0%) | |
| **HVLT-R DR** | DL3 | 3 | 1 (33%) | 1 | 1/5 | | (20%) | 0 | | 1 | | 0 |  |  |  |
| **HVLT-R RECOG** | DL3 | 3 | 1 (33%) | 1 | 1/5 | | (20%) | 1 | | 0 | | 0 |  |  |  |
| **TMTA** | DL3 | 3 | 1 (33%) | 1 | 1/5 | | (20%) | 1 | | 0 | | 0 |  |  |  |
| **TMTB** | DL3 | 3 | 0 (0 %) | 0 | 0/5 | | (0%) | 0 | | 0 | | 0 |  |  |  |
| **COWA** | DL3 | 3 | 0 (0 %) | 0 | 0/5 | | (0%) | 0 | | 0 | | 0 |  |  |  |
| **CTB COMP** | DL3 | 3 | 0 (0 %) | 0 | 0/5 | | (0%) | 0 | | 0 | | 0 |  |  |  |

***Reliable Change Index (RCI)-defined Decline****:* The frequency of RCI-defined Decline per test by dose level while on treatment are summarized in Table 5. The nature and pattern of RCI-defined Decline events paralleled the NCAE events. Of the 49 RCI-defined Decline events during treatment there was no further follow up testing to evaluate persistence or resolution of the RCI-defined Decline events in 22 cases. In the other 27 cases, 16 improved on follow up testing and 11 exhibited a persistent RCI-defined Decline event.

**Supplementary Table 5. Frequency of RCI-defined Decline Events by Dose Level While on Treatment.**

|  | **Arm** | **n** | **Patients with RCI-Decline Events**  **n (%)** | **Total RCI-Decline Events**  **n** | **Proportion of Visits with RCI-Decline Events**  **n (%)** | |
| --- | --- | --- | --- | --- | --- | --- |
| **HVLT-R TR** | DL1 | 7 | 3 (43%) | 5 | 5/26 | (19%) |
| **HVLT-R DR** | DL1 | 7 | 2 (29%) | 4 | 4/26 | (15%) |
| **HVLT-R RECOG** | DL1 | 7 | 4 (57%) | 9 | 9/26 | (35%) |
| **TMTA** | DL1 | 6 | 4 (67%) | 4 | 4/12 | (15%) |
| **TMTB** | DL1 | 6 | 3 (50%) | 4 | 4/25 | (16%) |
| **COWA** | DL1 | 7 | 0 (0%) | 0 | 0/26 | (0%) |
| **HVLT-R TR** | DL2 | 6 | 4 (67%) | 4 | 4/9 | (44%) |
| **HVLT-R DR** | DL2 | 6 | 5 (83%) | 5 | 5/9 | (1%) |
| **HVLT-R RECOG** | DL2 | 6 | 4 (67%) | 4 | 4/9 | (44%) |
| **TMTA** | DL2 | 6 | 3 (50%) | 3 | 3/9 | (33%) |
| **TMTB** | DL2 | 6 | 4 (67%) | 4 | 4/9 | (44%) |
| **COWA** | DL2 | 6 | 1 (17%) | 1 | 1/9 | (11%) |
| **HVLT-R TR** | DL3 | 3 | 0 (0%) | 0 | 0/5 | (0%) |
| **HVLT-R DR** | DL3 | 3 | 1 (33%) | 1 | 1/5 | (20%) |
| **HVLT-R RECOG** | DL3 | 3 | 1 (33%) | 1 | 1/5 | (20%) |
| **TMTA** | DL3 | 3 | 0 (0%) | 0 | 0/5 | (0%) |
| **TMTB** | DL3 | 3 | 0 (0%) | 0 | 0/5 | (0%) |
| **COWA** | DL3 | 3 | 0 (0%) | 0 | 0/5 | (0%) |

Note: Proportion of visits with RCI-Decline Events: (Total RCI-Decline Events / Total number of NC tests evaluated)
